# Ecology of trading strategies in a forex market for limit and market orders

**DOI:** 10.1371/journal.pone.0208332

**Published:** 2018-12-17

**Authors:** Takumi Sueshige, Kiyoshi Kanazawa, Hideki Takayasu, Misako Takayasu

**Affiliations:** 1 Department of Mathematical and Computing Science, School of Computing, Tokyo Institute of Technology, Nagatsuta-cho, Midori-ku, Yokohama, Japan; 2 Institute of Innovative Research, Tokyo Institute of Technology, Nagatsuta-cho, Yokohama, Japan; 3 Sony Computer Science Laboratories, Higashi-Gotanda, Shinagawa-ku, Tokyo, Japan; University of Warwick, UNITED KINGDOM

## Abstract

There is a growing interest to understand financial markets as ecological systems, where the variety of trading strategies correspond to that of biological species. For this purpose, transaction data for individual traders are studied recently as empirical analyses. However, there are few empirical studies addressing how traders submit limit and market order at the level of individual traders. Since limit and market orders are key ingredients finally leading to transactions, it would be necessary to understand what kind of strategies are actually employed among traders before making transactions. Here we demonstrate the variety of limit-order and market-order strategies and show their roles in the financial markets from an ecological perspective. We find these trading strategies can be well-characterized by their response pattern to historical price changes. By applying a clustering analysis, we provide an overall picture of trading strategies as an ecological matrix, illustrating that liquidity consumers are likely to exhibit high trading performances compared with liquidity providers. Furthermore, we reveal both high-frequency traders (HFTs) and low-frequency traders (LFTs) exhibit high trading performance, despite the difference in their trading styles; HFTs attempt to maximize their trading efficiency by reducing risk, whereas LFTs make their profit by taking risk.

## 1 Introduction

In financial markets, it has become possible to track trading of individual traders in detail mainly due to technological development. Such technical advances have invoked the curiosity of researchers to reveal mechanisms behind the deviation of actual financial markets from pure random processes, particularly in terms of the variety of trading strategies. Indeed, there is a growing interest in the empirical investigation on the variety of trading styles on transaction timing and frequencies. For example, the relationship between past average returns and trader’s decision to buy or sell stocks is reported in Refs. [1, 2]. The bilinear relationship was established between the average log turnover and the average log-account values in Ref. [3]. Reference [4] demonstrates that the response pattern to endogenous factors (price returns and volatility) and exogenous factors (the number of news and the sentiment created by news) can be classified by traders’ employment sectors using the liner-regression and partial correlation analysis. Network analyses in Refs. [5, 6] respectively revealed the synchronization in the trading activity among clusters and the time evolution of the networks and their roles in financial markets.

This research stream has been forming the field of the *market ecology*, where the variety of trading strategies corresponds to that of biological species. Indeed, Farmer stated in Ref. [7, 8] that the ecological concepts, such as the predator-prey relation, would be useful in understanding financial market microstructure. Whereas daily transaction data have been utilized for ecological studies of financial markets, however, the strategies of limit and market orders with a timescale of milliseconds are not studied in detail. Here, a limit order is used to specify the price at which the trader is willing to transact in future, and a market order is used to show a will to buy or sell the currency instantaneously. Since both limit and market orders are frequently issued between a transaction, the strategies for these two types of orders would include information on decision making process of traders, which we believe is a key to understand the market ecology.

**Fig 1:**
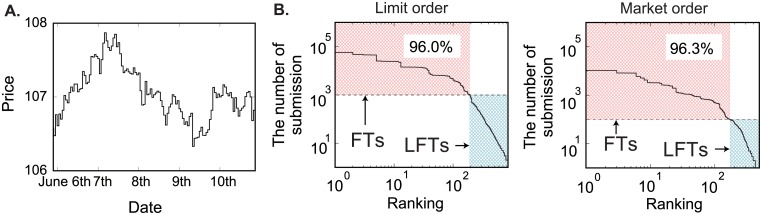
Definition of HFTs and LFTs. **A**, Transaction-price trajectory plotted every hour during the week starting from 6th June 2016. **B**, The number of submissions for limit and market orders. The ranking of traders (abscissa) is defined as the increase in number of submissions. Traders issuing more than 1000 (100) limit (market) orders are designated HFTs (in red) for the week; the others are defined as LFTs (in blue).

In this paper, we present a detailed report on the strategies of the limit and market orders of real traders in a forex market by tracking anonymously all individuals (1015 traders). The time and price precision of our dataset are millisecond and 0.005 JPY, respectively. We quantitatively characterized and classified their strategies to show their relation with market liquidity and trading performances, using the high-frequency data provided by Electronic Broking Services (EBS) in the dollar-yen currency market for the week from June 5th to June 10th, 2016. The trajectory of transaction prices every one hour are depicted in Fig 1(A). As seen from this figure, the prices are moderately fluctuating within a narrow price range without any bubbles or crashes, and seems to be appropriate to analyze usual limit-order and market-order strategies. The minimum volume unit for submission is one million dollars, and the total data-record and transactions were about 300 million and 68 billion dollars, respectively. We define the minimum price unit as a tenth pip (tpip, 0.001 yen) and the tick-time as an integer incremented by every transaction. Since the previous work of a EBS researcher [9] classified traders according to their submission frequencies, we define traders issuing not less than 1000 (100) limit (market) orders as FTs in this paper; they cover more than 95% of both orders (Fig 1(B)). The remaining traders are defined as low frequency traders (LFTs). The detailed strategic characteristics of FTs are examined from hereon to show derivation of the final overall ecological properties for all traders.

## 2 Methods

### 2.1 Short review of our analysis

To identify the trading strategies, we focused on the response pattern of limit orders and market orders to historical trends. We first introduced the coarse-grained tick intervals to calculate market price changes and the time lags to measure trends. To find the optimal parameters for both the tick intervals and the time lags, we then employed the multi-linear regression analysis for limit orders (see 2.2) and the multi-logistic analysis for market orders (see 2.4), respectively. In the following subsections, we described the detailed methodology of the identification of the optimal parameters.

### 2.2 Limit-order analysis

**Fig 2:**
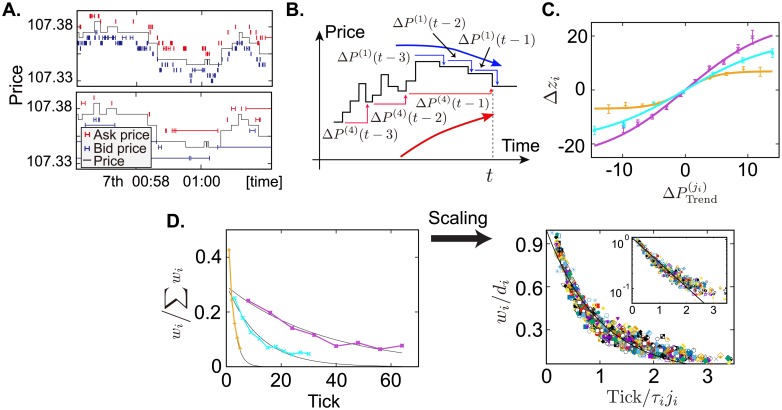
Determining timescales of the response to trends, and validation test results. **A**, Sample trajectories of limit orders issued by two FTs during six minutes: the lifetimes of ask and bid orders (red and blue lines, respectively), and a trajectory of transaction prices (black line). **B**, Schematic of the difference interpretation of trends from a single price trajectory. If a trader sees short-term price changes, the prices are in a down-trend (the blue curved arrow), whereas if a trader sees longer-term price changes, prices are in an up-trend (the red curved arrow). Different timescales lead to the different interpretations to historical trends. **C**, Relationship between the limit-order price change and trends. The historical periods over which to take an average are 1 tick (orange), 3 ticks (light-blue), and 8 ticks (violet). The hyperbolic tangent relation between them, empirically shown in early works [10, 11] focusing on the last single tick price change, also establishes price changes over several ticks. **D**, Three sample-normalized weights of regressors obtained by Eq (2) (left side) and the weights of 161 FTs after scaling (right side). As the three sample weights are well-approximated by exponential functions, we scale all FTs’ weights by scaling function *d*_*i*_ exp(−*k*/*τ*_*i*_). The right-hand-side graph shows about 85% of target traders (161 of 191 traders) determine trends by the exponential moving average. The inset plots the scaled weights on a log scale. Although there are deviations around the distribution tail, the overall trend is well captured by the exponential function.

We first quantified the timescale of trend-following behavior of each trader by studying the correlation between historical price trends and future limit-order price changes by traders. Let us look at the two sample trajectories of limit orders issued by two different traders, which illustrate the variety of the limit-order response speed to the change of transaction prices (Fig 2(A)). To quantify such heterogeneity in response timescales, we introduced a coarse-grained tick interval to calculate the market price changes and the maximum time lag up to which a trader refers in his/her memory in Fig 2(B). For example, let us compare the interpretation based on the blue and red lines in Fig 2(B). The blue line is based on 3 maximum time lag with 1 tick coarse-graining and indicates downward trends. On the other hand, the red line is based on 3 maximum time lag with 4 tick coarse-graining and indicates upward trends though the given transaction time series is the same. It is therefore necessary to determine (i) the timescale for coarse-graining and (ii) the maximum time lag for each trader.

In this paper, we determined such strategy parameters by maximizing the correlation between the historical market price changes and the future limit-order price changes of the trader. The historical price change calculated according to *j*_*i*_ tick coarse-graining and *k*-th time lag is

$$\Delta P_{\text{Trend}}^{\left(j_{i}\right)}\left(t-k\right)\equiv P\left(t-j_{i}\left(k-1\right)\right)-P\left(t-j_{i}k\right),$$ (1)

and the limit-order price change for *i*-th trader is given by Δ*z*_*i*_(*t*) ≡ *z*_*i*_(*t* + 1) − *z*_*i*_(*t*). Here $z_{i}\left(t\right)\equiv \frac{1}{2}\left(z_{i}^{+}\left(t\right)+z_{i}^{-}\left(t\right)\right)$ is the mid-price of the best ask price $z_{i}^{+}\left(t\right)$ and the best bid price $z_{i}^{-}\left(t\right)$. *P*(*t*) is the transaction price at time *t*. When there is no bid (ask) quote, $z_{i}^{-}\left(t\right)$ ($z_{i}^{+}\left(t\right)$) is substituted by the last bid (ask) quote price, and extreme limit-order price changes more than 100 tpip are excluded from the following analysis. We showed the average changes of Δ*z*_*i*_(*t*) conditional on the historical price changes $\Delta P_{\text{Trend}}^{\left(j_{i}\right)}\left(t\right)$ in Fig 2(C). In this figure, we employed Δ*z*_*i*_(*t*) of the 6th trader (orange), the 65th trader (light-blue), and the 180th trader (violet), respectively. Correspondingly, $\Delta P_{\text{Trend}}^{\left(j_{i}\right)}\left(t\right)$ is calculated on the basis of the 1 tick coarse-graining and 5 maximum time lags (orange), the 3 tick coarse-graining and 10 maximum time lags (light-blue), and the 8 tick coarse-graining and 8 maximum time lags (violet). We found that these three examples can be well-approximated by the hyperbolic tangent curves denoted by the black line. It is worth noting that this relationship is a straightforward generalization of the formula found in Refs. [10, 11]. In their paper, they found the special case of this relationship by fixing *j*_*i*_ = 1 such that $\Delta z_{i}\left(t\right)=c_{i}\tanh\left(\Delta P_{\text{Trend}}^{\left(1\right)}\left(t\right)/p_{i}^{*}\right)$ where $c_{i},p_{i}^{*}$ are constants.

Given this result, the general relationship between Δ*z*_*i*_(*t*) and $\Delta P_{\text{Trend}}^{\left(j_{i}\right)}\left(t\right)$ can be formulated by

$$\Delta z_{i}\left(t\right)=c_{i}\tanh(\Delta P_{\text{Trend}}^{\left(j_{i}\right)}\left(t\right)+\alpha_{i}),$$ (2)

$$\Delta P_{\text{Trend}}^{\left(j_{i}\right)}\left(t\right)\equiv \sum_{k=1}^{K_{i}}w_{i}\left(k\right)\Delta P^{\left(j_{i}\right)}\left(t-k\right)+\sigma \epsilon_{i}\left(t\right),$$ (3)

where *K*_*i*_ is the number of time lags, *w*_*i*_(*k*) is the coefficient of regressors at time *k*, *ϵ*_*i*_(*t*) is the white noise, and *α*_*i*_, *σ* are constants.

On the basis of this relation, we retroactively incremented the number of time lags under multiple time-coarse-graining and optimize the parameter set to maximize the correlation between the historical market price change and the future quoted price of a trader. Parameters *α*_*i*_ and *w*_*i*_(*k*) are estimated through the multi-linear regression analysis, and the trend-following strength *c*_*i*_ and the maximum time lag *K*_*i*_ are determined by another process explained in 2.3. The multi-linear regression analysis was performed only when Δ*z*_*i*_(*t*) ≠ 0. We reduced this non-linear equation to a linear equation using the inverse function of hyperbolic tangent and then performed a multi-linear regression analysis.

We next found that coefficients *w*_*i*_(*k*) approximately decays exponentially, whereby the characteristic timescale of trend-following can be defined by the decay timescale in Fig 2(D). After determining the maximum time lag *K*_*i*_ and the time-coarse-graining such that the adjusted coefficient of determination ($R_{i,\text{adj}}^{2}\equiv 1-\left(1-R_{i}^{2}\right)\left(N_{i}-1\right)/\left(N_{i}-K_{i}-1\right)$) takes a maximum, we show three examples of coefficients of the regressors with the approximate exponential functions (Fig 2(D)). $R_{i}^{2}$ is the coefficient of determination, and *N*_*i*_ is the number of samples. We found the typical feature of the coefficients for the three traders (the 6th, the 65th, and the 180th) is their exponential form,

$$w_{i}\left(k\right)=d_{i}\exp(-\frac{k}{\tau_{i}}),$$ (4)

with various time constants *τ*_*i*_ and heights *d*_*i*_, at least for the body part of the coefficient. This finding is established not only for these three traders, but also for 161 traders as illustrated by the scaling *w*_*i*_/*d*_*i*_ (Fig 2(D)), where the body of the distributions approximately collapse onto the exponential master curve despite several deviations especially around its tail. We note that we could not identify the function form for the tail in the absence of sufficient number of data points. Indeed, the typical maximum time lag is five and is not sufficient to conclude whether the true tail obeys other functions such as a power-law tail or not. Fortunately, however, the body part of the weight function is the most important to measure trends and thus we employed the exponential fitting function for simplicity in this paper.

This result shows the direct evidence that the EMA is a typical metrics to measure market price trends [12]. We excluded from these plots data of traders for which the sum of the squared errors (SSE) of the prediction normalized by the *d*_*i*_ exceeds the 0.1 as the fitting threshold. Eleven traders were excluded from the EMA trend-followers and classified among the non-EMA trend-following cluster. We define the reference time for the *i*th trader as a product of the optimal tick interval *j*_*i*_ with the estimated time constant *τ*_*i*_. If there is only one data point satisfying the significant level for the correlation, we skip the fitting procedure and assume *τ*_*i*_ = 1.

### 2.3 Determination of the trend-following strength *c*_*i*_ and the maximum time lag *K*_*i*_ in Eqs (2) and (3)

We explain the way to determine both *c*_*i*_ and *K*_*i*_ introduced in Eqs (2) and (3). We performed the following iteration method with a given coarse-graining time interval *j* ranging from 1 to 20 ticks.

P1. Set *c*_*i*_ = 5 and ${\tilde{K}}_{i}=1$ as initial values. Here ${\tilde{K}}_{i}$ is a candidate of *K*_*i*_.
P2. Calculate $\Delta \tilde{z}\left(t\right)\equiv \tanh^{-1}\left(\Delta z_{i}\left(t\right)/c_{i}\right)$.
P3. Perform the multi-linear regression analysis for the timeseries of $\Delta \tilde{z}\left(t\right)$ as a regressand, and those of $\Delta P_{\text{Trend}}^{\left(j_{i}\right)}\left(t\right)$ as regressors.
P4. If the *p*-value of the coefficient obtained above is lower than the threshold, we consider the obtained coefficient as statistically significant. We then increment ${\tilde{K}}_{i}$ and iterate this process until ${\tilde{K}}_{i}=20$. See Ref. [13] for the calculation of the *p*-value. In this analysis, we employ 0.001 for the threshold of the *p*-value, which is also employed in Ref. [14].
P5. Once the last *p*-value of the coefficient is higher than the threshold, we stop this iteration process and set $K_{i}=\tilde{K_{i}}-1$.
P6. Calculate the relationship between the average limit-order price changes and trends on the basis of the obtained *w*_*i*_ and *K*_*i*_, and then calculate a new *c*_*i*_ by fitting the hyperbolic tangent function as well as $R_{i,\text{adj}}^{2}$.
P7. When the relative difference between the current and previous *c*_*i*_ is larger than 1%, we repeat back to the process P2. Otherwise, the iteration process is terminated for the given *j*. We repeat this fitting of *c*_*i*_ 100 times for convergence. Note that, in this study, three traders were classified to the non EMA trend-following cluster since their *c*_*i*_ did not converge for all coarse-graining time intervals.

### 2.4 Market-order analysis

**Fig 3:**
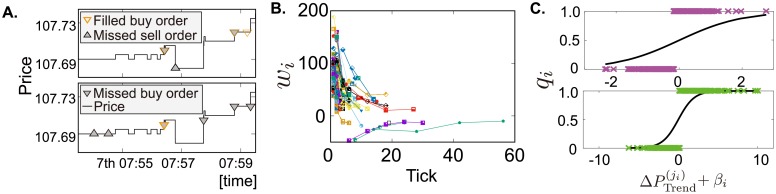
Determining timescales of the response to trends, and validation test results. **A**, Sample trajectories of market orders by two FTs during six minutes. Upward (downward) triangles are sell (buy) orders. The colour of triangles represents the status of market orders: orange (gray) signifying filled (missed). The black line is the trajectory of transaction prices. **B**, Weights *w*_*i*_ in Eq (3) of 131 traders obtained from a logistic analysis Eq (5) are plotted at *j*_*i*_*k*. To obtain the optimal *w*_*i*_, we follow the same procedure with the limit-order analysis except for using the SSE, not $R_{i,\text{adj}}^{2}$. **C**, The horizontal and vertical axis mean the historical trends and the probabilities controlling the direction of market orders, respectively. The black is the standard logistic function. On top of that, we depict the magnitude of the historical trends for two traders as cross-marks, which is obtained by the Eq (5). The market orders issued to the buy (sell) side are depicted by the cross-marks at 1 (0).

We describe how to determine the time interval referred to by traders in making a decision to issue market orders. Sample data points for which market orders are issued by two real traders were plotted (Fig 3(A)), and traders also seemingly have different responses to price trends due to the similar reason explained in limit-order analysis. Note that traders are allowed to attach the acceptable transaction price to market orders. If the current best price is worse than that price, a market order fails. To analyze how traders respond to trends, we used logistic regression in the parallel method to analyze limit orders.

To determine the time-coarse-graining, we use a similar analytic method to the aforementioned limit order analysis, based on the multi-logistic regression instead of the multi-linear regression:

$$\log(\frac{q_{i}\left(t+1\right)}{1-q_{i}\left(t+1\right)})=\Delta P_{\text{Trend}}^{\left(j_{i}\right)}\left(t\right)+\beta_{i}+\epsilon_{i}\left(t\right)$$ (5)

where *q*_*i*_(*t* + 1) and 1 − *q*_*i*_(*t* + 1) show the probabilities for buying orders and for selling orders when issuing market orders, and *β*_*i*_ is a constant parameter; the notation for other parameters is the same as in the limit-order analysis. We set the threshold of the *p*-value at 0.05 for this market order analysis, which is smaller than that for the limit order analysis since the number of limit orders is approximately ten times larger than that of market orders (Fig 1(B)). Note that despite the weaker threshold employed in this section, this criteria is generally accepted in the field of statistics [13].

After determining both the time-coarse-graining and the maximum time lag for each trader, we plotted the coefficients obtained by the multi-logistic regression for 131 traders (Fig 3(B)). Most of the coefficients are positive, but a few are negative. We classify their strategies based on the sign of $\bar{w_{i}}\equiv \sum_{k=1}^{K_{i}}w_{i}\left(k\right)/K_{i}$.

We next show the fitting result based on our logistic regression method. The horizontal and vertical axis of Fig 3(C) respectively indicate the historical trends and the probabilities controlling the direction of market orders (i.e. buy or sell). The black line in this figure is the standard logistic function. In addition, we marked the magnitude of historical trends as cross-marks for two traders when market orders are issued, which are calculated according to Eq (5). The market orders issued to the buy (sell) side are depicted by the cross-marks at 1 (0). Given the vertical axis showing the probabilities controlling the direction of market orders, the top (bottom) graph shows a trader weakly (strongly) motivated by historical trends.

## 3 Results and discussion

### 3.1 Clustering of limit-order strategies

To understand financial markets as a market ecology, we are interested in the typical differences of limit-order strategies, rather than the detailed differences of them in this paper. We thus cluster the limit-order strategies by the similarity of trend-following timescales, and then track the differences of the limit-order activities back to the differences of their limit-order book shapes, which has been a topic of study of late [10, 11, 15–19].

**Fig 4:**
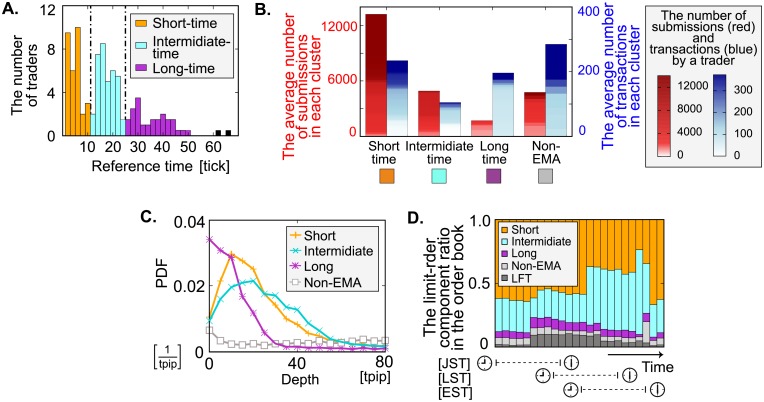
Analysis of limit-order strategies. **A**, Distribution of the trend-following reference time of 161 FTs. There are three typical clusters ranging from 1 tick to 10 ticks (short-time cluster, marked in orange), from 11 ticks to 23 ticks (intermediate-time cluster, in light-blue), and from 24 ticks to 50 ticks (long-time cluster, in violet), all which are obtained using the *k*-means method. They typically correspond to half, three, and six minutes given the average transaction interval is 9 seconds in this week. Two samples around 60 ticks were excluded as exceptions. **B**, The average number of limit orders (red) and transactions as limit orders (blue) for each cluster. The gradations in the plot bars presents a heat map of the ascending number of limit orders and that of transactions as limit orders by a trader in each cluster. The short-time (long-time) trend-followers submit the most (least) frequently, whereas the number of transactions for intermediate-time trend-followers is least despite a relatively large number of submissions. **C**, Probability density functions of the limit-order distributions (PDFs) conditional on the limit-order strategies. The peak of PDF of intermediate-time trend-followers lies far behind the best prices compared with other trend-followers, which reduce the transaction frequencies of intermediate-time trend-followers. **D**, Time-series of the ratio for the number of limit orders in the order book issued by each cluster. Each bar represents the hourly average ratio. JST, LST, and EST signify Japan, London, and Eastern Standard Times, respectively. The clock in the figure starts from 9:00 am to 6:00 pm for each standard time. Dark-gray bars represent the fraction of limit orders issued by LFTs.

Fig 4(A) shows the distribution of the reference times. Using the *k*-means method, we classified the reference times into three clusters: the short-time (typically 4 ticks; 30 sec), intermediate-time (typically 20 ticks; 2.5 min), and long-time (typically 40 ticks; 5 min) clusters. To determine the cluster size, we employ the silhouette method [20] and compared clusters ranging from size 2 to 5. We conclude that three clusters form an optimal size in terms of both the silhouette coefficient and the thickness of clusters. Note that all FTs were classified as either EMA trend-followers or non-EMA trend-followers.

What does this timescale difference imply? To answer this question, we studied the average number of limit-order submissions and that of transactions as limit orders for each cluster (Fig 4(B)). Although the number of submissions has a trivial correlation in that short-time (long-time) trend-followers submit the most (least) frequently, the number of transactions has a nontrivial correlation; the number of transactions for intermediate-time trend-followers is least despite a relatively large number of submissions. To investigate this nontrivial correlation, we studied the limit order book shape for each cluster, representing the typical depth of order placements (Fig 4(C)). These order-book profiles provide clear answers to the nontrivial behaviour. The short-time and long-time trend-followers maintain their orders near the best prices, leading to frequent transactions. The non-EMA trend-followers also transact frequently because they leave their orders without price modifications. However, the intermediate-time trend-followers maintain their orders relatively far from the best prices compared with other trend-followers and therefore are less likely to transact.

We remark on the intraday pattern of limit-order strategies. Fig 4(D) is the hourly limit-order component ratio in the order book. In Tokyo, trend-following of short duration is the dominant strategy during the daytime, whereas in New York it is of intermediate duration. Given the order-book shape in Fig 4(C), Tokyo (New York) traders are bullish (bearish) on transactions at current best prices in the daytime.

### 3.2 Clustering of market-order strategies

**Fig 5:**
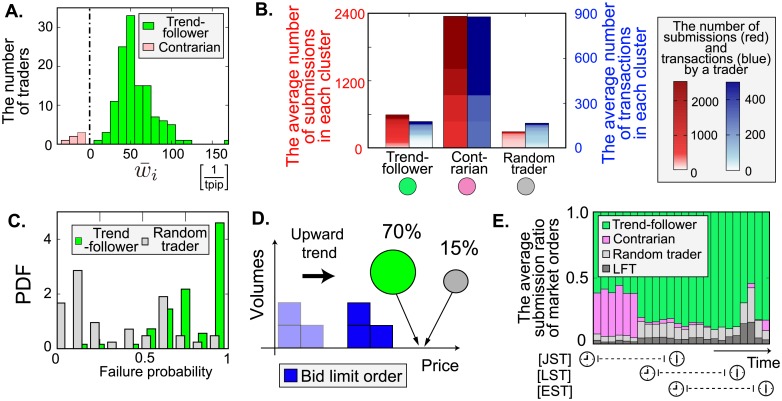
Analysis of market-order strategies. **A**, Distribution of ${\bar{w}}_{i}$ quantifying the average strength of trend-following for market orders. The original samples *w*_*i*_ are shown in Fig 4. A positive (negative) ${\bar{w}}_{i}$ denotes a *i*th trader is a trend-follower (contrarian), and represented by a green (pink) plot bar. **B**, Average number of market orders (red) and that of transactions as market orders (blue) for each cluster. The gradation in plot bars presents a heat map of the ascending number of market orders and that of transactions as market orders by a trader in each cluster. Contrarians are active despite their small size. **C,D**, Failure probabilities of market orders in transactions (C) and the probabilities in which market orders are issued at prices better than the current best prices (D). The green (gray) bars and circles represent the strategic properties of trend-followers (random traders). The median failure probability of trend-followers (random traders) is 83% (24%), and the probability of trend-followers issuing market orders at better prices than the current best price is 70% (15%). Trend-follower may be attempting to obtain better prices than current best prices by submitting market orders in advance. **E**, Time-series of the ratio for the number of market orders issued by each cluster. Each bar represents the hourly average ratio. The dark-gray bars signify the fraction of market orders issued by LFTs.

We report the detail properties of market-order strategies. Fig 5(A) is the distribution of market-order strategies of FTs, which is quantified by ${\bar{w}}_{i}$: positive (negative) ${\bar{w}}_{i}$ implies that the *i*th trader is a trend-follower (contrarian), who issues buy orders during positive (negative) trends, and sell orders during negative (positive) trends. In our market-order analysis, we found several FTs were contrarians but most were trend-followers. Note that traders showing no significant correlation with trends were classified within the random cluster.

To extract features of the strategies, we studied the number of market orders and that of transactions as market orders for each cluster (Fig 5(B)). We found that the contrarians are overwhelmingly active despite their small size. Indeed, the first and second most frequent traders were contrarians in our dataset. Notably, a previous study [14] reports the existence of contrarians at the trader group level.

Another feature is the difference in the degree of contributions to transactions. Given the large number of market orders trend-followers issue, the transaction count is relatively small compared with that for random traders. To clarify this imbalance, we defined the failure probability as the fraction of failed market orders to total market orders (see S1 Appendix). Fig 5(C) shows that the typical failure probability of the trend-followers (84%) is almost four times higher than that of the random traders (23%). Why did trend-followers submit such “meaningless” market orders? One of our conjectures is that trend-followers may aim the latency during price-matching processes (we have another conjecture based on pinging strategies [14, 21–24] which is illustrated in S2 Appendix). Given this latency, a good strategy may be to hit in advance better prices than the current best prices following their trend prediction. Indeed, as illustrated in Fig 4(D), most of the market orders by trend-followers were issued at better prices (70%) than the current best prices compared with random traders (15%), a practice commonly observed during the week (Fig 4(E)). Note that the individual Tokyo traders in the daytime behave as contrarians, which is consistent with a previous work [25] indicating that contrarian behaviour is the favoured and profitable strategy in Japan.

### 3.3 Strategy-matrix analysis

**Fig 6:**
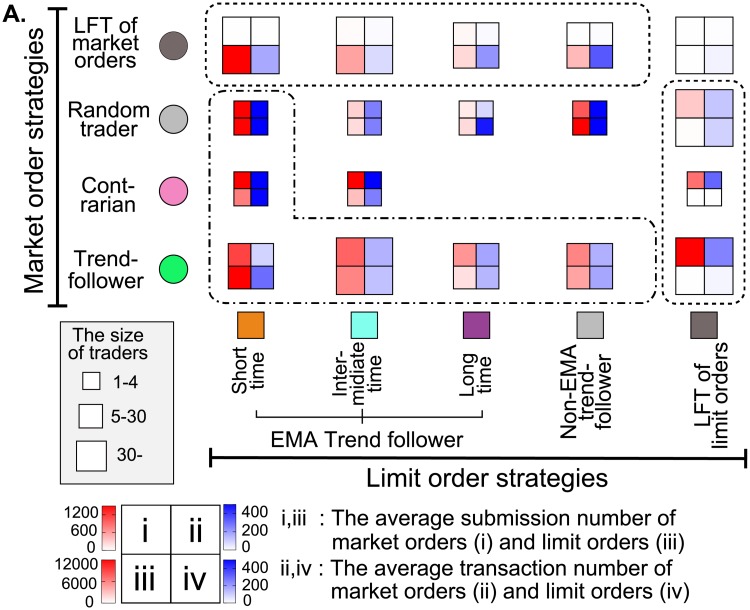
Illustrating the overall strategic properties as an ecological matrix. **A**, The frequencies of order submissions and that of transactions issued by traders employing strategies using various combinations of limit and market orders. The box size represents the number of traders. The blank elements indicates the absence of traders adopting corresponding combination strategies. We confirm (i) the immense contributions to submissions and transactions by the short-time cluster for limit order strategies and the trend-follower cluster for market-order strategies surrounded by a chain line, and (ii) a large population of traders tend to mainly submit either limit orders or market orders given the size of boxes surrounded by a dotted line.

Finally, as a strategy-analysis summary including LFTs, Fig 6(A) shows an ecological matrix (4 × 5 elements with two empty elements) illustrating the number of traders, submissions, and transactions for the combination strategies of both order types. This figure shows the following two characteristics: one is the immense contribution to submissions and transactions by the short-time trend-followers for limit orders and the trend-followers for market orders (i.e., the clusters surrounded by the chain line). They occupy 44% (40%) of submissions as limit (market) orders and 28% (35%) of transactions as limit (market) orders, though their population is relatively small (80 traders, 7.8% of all population). The other characteristic is the tendency that there are many traders who submit mainly either limit or market orders (i.e., the cluster surrounded by the dot line; 189 traders, 19% of all population). This characteristic implies that they might be specialized in either limit or market order strategy.

**Fig 7:**
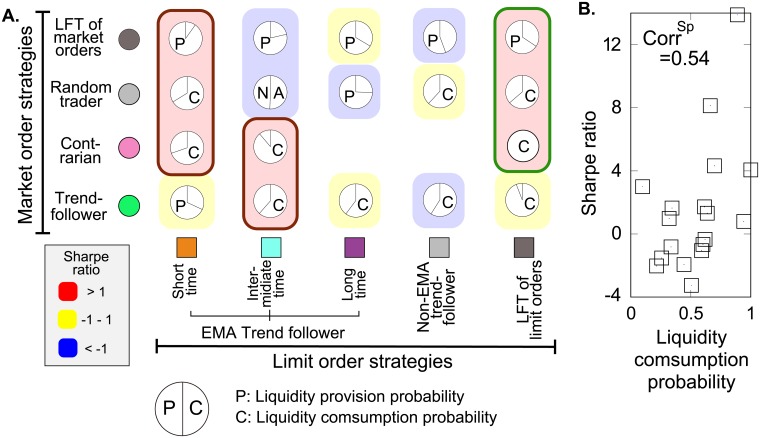
Illustrating the trading performances as an ecological matrix. **A** The pie charts quantify the overall balance between the liquidity provision and consumption of the cluster. Here the liquidity provision (consumption) is measured as the total volumes transacted as makers (takers). Each cluster is classified as either a liquidity provider or consumer through a statistical test on the significance of the imbalance between liquidity provision (P) and consumption (C). N/A signifies that the imbalance is not statistically significant. In addition, clusters are colour coded (red, yellow, or blue) to mark their trading performances as measured by the Sharpe ratio. The breakdown of trading performances and trading profits of clusters the high performance clusters coded by a brown and green line are further investigated in Fig 8. **B**, Scattering plot between the liquidity consumption ratios and the Sharpe ratios with Spearman’s rank correlation coefficient. This positive correlation implies that more frequently traders transact as takers, better performances traders are likely to exhibit.

We further investigate the relationship between the limit-order and market-order strategies with (i) trader’s role for liquidity and (ii) their trading performances in Fig 7(A). We show pie charts quantifying the overall balance between liquidity providers and consumers. Each component is highlighted to illustrate trading performances as measured by the Sharpe ratio (see S3 Appendix). As one may notice, there exists the strong correlation between the Sharpe ratios and liquidity consumption probabilities (0.54 as measured by the Spearman rank correlation (Fig 7(B))). This correlation suggests the traders consuming (providing) the liquidity are likely to exhibit good (bad) trading performances as they take on risk for (not for) their sake. This result is consistent with the analysis concerning the inventory risk for liquidity providers to the decline in asset prices [26].

**Fig 8:**
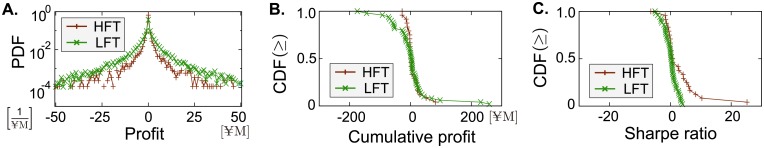
Illustrating the profit distributions and the trading performances of HFTs and LFTs. **A,B,C** Trading profits and trading performances of banks, the traders in the high-performance cluster of which are aggregated. We exclude from the aggregation traders with transaction counts below 100 in the week. Specifically, (A)–(C) plots the trading profit distributions per trader calculated every 20 minutes during a week, the cumulative profit distributions at the end of the week, and the distribution of the trading performances measured by the Sharpe ratio.

It would be interesting to explore why the two opposite types of clusters surrounded by a brown line (typically high frequency traders (HFTs)) and a green line (LFTs) in Fig 7 exhibit high trading performances. We therefore provide the breakdown properties of these two clusters as a case study. After aggregating traders at the bank level, we plotted the distributions of trading profits calculated every 20 minutes, the total trading profits in this week, and the Sharpe ratios (Fig 8(A), 8(B) and 8(C), respectively). From these figures, the maximum profit (trading efficiency) of HFTs is smaller (larger) than that of LFTs, indicating HFTs (LFTs) make small (large) profits using small (large) inventory at stake. Given the previous study highlighting that HFTs are highly profitable by taking advantage of response speed, this result indicates counterintuitively that strategies of HFTs and LFTs seem equilibrium-balanced by optimization according to different metrics at least in our dataset.

## 4 Conclusion

In summary, focusing on the historical market trends, we classified the timescale of the limit-order trend-following and the response pattern for market-order strategy to the trends. The differences in the timescale of the limit-order trend-following are closely related to the limit-order book shape. The traders with the short and long trend-following timescales are bullish to transact with the current best price, while traders with intermediate time are bearish. The differences in the response pattern of market orders to trends have a close link to failure probabilities; how many market orders are finally transacted out of all submitted market orders. The failure probabilities of trend-followers are quite high while those of random traders are low. This difference may imply that trend-followers predict the current trends and submit market orders to the price better than current best prices in advance, whereas random traders have no prediction to the trends and do not behave in this way.

We further calculated the submission and transaction frequency matrix combined with both limit and market order’s strategies. This matrix illustrates two interesting properties; one is the strong correlation between the Sharpe ratios and liquidity consumption probabilities and the other is the fact that both HFTs and LFTs recorded high-trading performance despite the difference of their trading styles.

Here we note that we cannot exclude the possibility that the result of our analysis might depend on the data period. Since the total period covered by our data set is five days, some of our analysis might depend on the specific economic conditions during this period. The characteristics of our data period is as follows: As shown in Fig 1(a), the overall price was moderately fluctuating within a narrow price range. As a financial event, there was a regular speech by Janet Louise Yellen on 6th June 2016. This speech might have impact on our analysis, thought it is evaluated to have a limited impact on forex markets [27]. It is an interesting future topic to apply our method to (i) volatile markets, and (ii) the markets just after the economic events in order to clarify how the market condition affects the strategies among traders.

We also note another possibility that our data analysis would be affected: *hidden orders*. To trade large quantities of orders, traders are prone to split these orders into small pieces to hide their true size, which is called hidden orders [28]. When traders handle hidden orders, they could switch their normal trading strategies to hidden order strategies in response to the actual trading demand. This strategy switching would affect our data analysis, and the extent of its impact on our data analysis is an interesting topic of future research.

To the best of our knowledge, this is the first systematic empirical report that clusters limit-order and market-order strategies from the level of individual traders and explains the several aspects of financial markets from a viewpoint of this clustering. This results give a robust foundation for the ecological perspective in financial markets and facilitate the construction of the ecological model to explain various complex phenomena such as market regulation, price stabilization, and risk mitigation. For example, it is of interest to evaluate which strategies facilitate the growth of bubbles or crashes, and predict the beginning of price bubbles or crashes via an increase in activity of these strategies.

## Supporting information

S1 Appendix Calculation method for the failure probability. (DOCX)

S2 Appendix Another conjecture for large failure probabilities: Aiming at trading with hidden orders. (DOCX)

S3 Appendix Calculation method of the Sharpe ratio. (DOCX)

## References

[1] Odean T. Are investors reluctant to realize their losses?. The Journal of finance. 1998;53: 1775–1798. 10.1111/0022-1082.00072

[2] Grinblatt M, & Keloharju M. The investment behavior and performance of various investor types: a study of Finland’s unique data set. Journal of financial economics. 2000;55: 43–67. 10.1016/S0304-405X(99)00044-6

[3] de Lachapelle DM, & Challet D. Turnover, account value and diversification of real traders: evidence of collective portfolio optimizing behavior. New Journal of Physics. 2010;12: 075039 10.1088/1367-2630/12/7/075039

[4] Lillo F, Miccichè S, Tumminello M, Piilo J, & Mantegna RN. How news affects the trading behaviour of different categories of investors in a financial market. Quantitative Finance. 2015;15: 213–229. 10.1080/14697688.2014.931593

[5] Tumminello M, Lillo F, Piilo J, & Mantegna RN. Identification of clusters of investors from their real trading activity in a financial market. New Journal of Physics. 2012;14: 013041 10.1088/1367-2630/14/1/013041

[6] Musciotto F, Marotta L, Piilo J, & Mantegna RN. Long-term ecology of investors in a financial market. Palgrave Communications. 2018;4: 92 10.1057/s41599-018-0145-1

[7] Farmer JD, Skouras S. An ecological perspective on the future of computer trading. Quantitative Finance. 2013;13: 325–346. 10.1080/14697688.2012.757636

[8] Farmer JD. Market force, ecology and evolution. Industrial and Corporate Change. 2002;11: 895–953. 10.1093/icc/11.5.895

[9] Schmidt A. Ecology of the modern institutional spot FX: The EBS market in 2011. SSRN. 2012; 1984070.

[10] Kanazawa K, Sueshige T, Takayasu H, Takayasu M. Derivation of the Boltzmann equation for financial Brownian motion: Direct observation of the collective motion of high-frequency traders. Physical Review Letter. 2018;120: 138301 10.1103/PhysRevLett.120.138301 10.1103/PhysRevLett.120.138301 29694225

[11] Kanazawa K, Sueshige T, Takayasu H, Takayasu M. Kinetic Theory for Financial Brownian Motion from Microscopic Dynamics. Physical Review E. 2018;98: 052317 10.1103/PhysRevE.98.052317 10.1103/PhysRevLett.120.138301 29694225

[12] Grebenkov DS, Serror J. Following a trend with an exponential moving average: Analytical results for a Gaussian model. Physica A. 2014;394: 288–303. 10.1016/j.physa.2013.10.007

[13] Hamilton JD. Time series analysis Vol. 2 Princeton. NJ: Princeton university press 1994.

[14] Brogaard J. High frequency trading and its impact on market quality. Northwestern University Kellogg School of Management Working Paper 66. 2010.

[15] Donier J, Bonart J, Mastromatteo I, Bouchaud JP. A fully consistent, minimal model for non-linear market impact. Quantitative Finance. 2015;15: 1109–1121. 10.1080/14697688.2015.1040056

[16] Tóth B, Lemperiere Y, Deremble C, De Lataillade J, Kockelkoren J, & Bouchaud JP. Anomalous price impact and the critical nature of liquidity in financial markets. Physical Review X 2011;1: 021006.

[17] Zhou WX. Universal price impact functions of individual trades in an order-driven market. Quantitative Finance. 2012;12: 1253–1263. 10.1080/14697688.2010.504733

[18] Bouchaud JP, Mézard M, Potters M. Statistical properties of stock order books: empirical results and models. Quantitative Finance. 2002;2: 251–256. 10.1088/1469-7688/2/4/301

[19] Yura Y, Takayasu H, Sornette D, Takayasu M. Financial brownian particle in the layered order-book fluid and fluctuation-dissipation relations. Physical Review Letter. 2014;112: 098703 10.1103/PhysRevLett.112.098703 10.1103/PhysRevLett.112.098703 24655287

[20] Rousseeuw PJ. Silhouettes: a graphical aid to the interpretation and validation of cluster analysis. Journal of computational and applied mathematics. 1987;20: 53–65. 10.1016/0377-0427(87)90125-7

[21] ebs-dealing-rules-general-terms-291015, Available at https://www.nexmarkets.com/products-and-services/market-regulation/rulebooks/ebs Cited 16 June 2018.

[22] Securities US, & Exchange Commission. Part III: Concept release on equity market structure; Proposed Rule, 17 CFR Part 242. Federal Register. 2010;75: 3594–3614.

[23] Boulatov A, George TJ. Hidden and displayed liquidity in securities markets with informed liquidity providers. The Review of Financial Studies. 2013;26: 2096–2137. 10.1093/rfs/hhs123

[24] Xie J. Criminal regulation of high frequency trading on China’s capital markets. International Journal of Law, Crime and Justice. 2016;47: 106–120. 10.1016/j.ijlcj.2016.09.002

[25] McInish TH, Ding DK, Pyun CS, Wongchoti U. Short-horizon contrarian and momentum strategies in Asian markets: An integrated analysis. International review of financial analysis. 2008;17: 312–329. 10.1016/j.irfa.2006.03.001

[26] Grossman SJ, Miller MH. Liquidity and market structure. Journal of Finance. 1988;43:617–633. 10.1111/j.1540-6261.1988.tb04594.x

[27] halo finance, Fed rate hikes on hold, https://www.halofinancial.com/news/currency-insights/june-2016/daily-currency-insight-7th-june-2016, Cited 17 September 2018.

[28] Moro E, Vicente J, Moyano LG, Gerig A, Farmer JD, Vaglica G, et al Market impact and trading profile of hidden orders in stock markets. Physical Review E. 2009;80: 066102 10.1103/PhysRevE.80.066102 10.1103/PhysRevE.80.066102 20365226

