# Supplementary material for: Ecology of trading strategies in a forex market for limit and market orders
Source: PLoS One. 2018 Dec 17;13(12):e0208332. doi: 10.1371/journal.pone.0208332 (PMC6296528; doi:10.1371/journal.pone.0208332)
Supplement: S1 Appendix — (DOCX) [file pone.0208332.s001.docx]

S1 Calculation method for the failure probability

In calculating the failure probability of market orders, defined as one minus the success probability, we focus on the number of market order submissions and of subsequent transactions, but do not take volumes into consideration.　For example, when a trader issues a market order with ten volumes and five are transacted, we count only one market order success in the single transaction.　The success probability, calculated as a ratio of the number of transactions　and market orders, is 1/1=1, and the failure probability therefore is 0.
