# Supplementary material for: Ecology of trading strategies in a forex market for limit and market orders
Source: PLoS One. 2018 Dec 17;13(12):e0208332. doi: 10.1371/journal.pone.0208332 (PMC6296528; doi:10.1371/journal.pone.0208332)
Supplement: S2 Appendix — (DOCX) [file pone.0208332.s002.docx]

S2 Another conjecture for large failure probabilities:

aiming at trading with hidden orders

We first explain the hidden orders in EBS markets.　For the benefit of human traders in EBS markets, they are allowed to attach a hidden aggressive dealable price range to limit orders (called a pip discretion [1]). If a new limit or market order is issued in this range, the limit order is transacted with them even though they have a worse price than the price when the human trader initially issued the limit order. This sort of hidden orders is currently in vogue in large financial markets such as New York Security Market and EBS Forex market.

These hidden orders may motivate traders to issue market orders at the price better than the current best prices as trading with these orders　leads to potential arbitrage opportunities.

We conjecture this mechanism is another reason for the large failure probabilities of market orders. This behavior in issuing orders to a place where no liquidity is supplied is known as a pinging [2] and is a traditional liquidity detection strategy anticipating to transact with undisplayed orders such as hidden orders. Pinging and undisplayed orders are currently quite common in various financial markets. Their impact on financial markets has been investigated [3-5].

[1] <https://www.nexmarkets.com/~/media/Files/E/EBS-Brokertec/rulebooks/ebs-dealing-rules-ebs-market-appendix-291015.pdf>, (Retrieved date: 3^rd^ December 2018)

[2] Securities, U. S., & Exchange Commission. Part III: Concept release on equity market structure; Proposed Rule, 17 CFR Part 242. Federal Register **75**, 3594-3614 (2010).

[3] Brogaard, J. High frequency trading and its impact on market quality. Northwestern University Kellogg School of Management Working Paper 66 (2010).

[4] Boulatov, A., & George, T. J. Hidden and displayed liquidity in securities markets with informed liquidity providers. *Rev. Financial Stud.* **26**, 2096-2137 (2013).

[5] Xie, J. Criminal regulation of high frequency trading on China's capital markets. *Int. J. Law Crime Justice* **47**, 106-120 (2016).
