# Supplementary material for: Ecology of trading strategies in a forex market for limit and market orders
Source: PLoS One. 2018 Dec 17;13(12):e0208332. doi: 10.1371/journal.pone.0208332 (PMC6296528; doi:10.1371/journal.pone.0208332)
Supplement: S3 Appendix — (DOCX) [file pone.0208332.s003.docx]

S3 Calculation method of the Sharpe ratio

The Sharpe ratio using return rates calculated every $u$-minute is obtained from

$$S_{i}^{(u)}=\frac{\bar{r}_{i}^{\left( u \right)}(t)}{\sigma_{i}^{(u)}},$$

$$\bar{r}_{i}^{(u)}\left( t \right)\equiv\frac{1}{N^{(u)}}\sum_{v=1}^{N^{(u)}} r_{i}^{(u)}\left( t+uv \right),$$

$$r_{i}^{\left( u \right)}(t)=\frac{s_{i}\left( t \right)-s_{i}\left( t-u \right)}{\bar{V}_{i}\left( t \right)},$$

$$\bar{V}_{i}\left( t \right)=\frac{V_{i}\left( t \right)+V_{i}\left( t-u \right)}{2}M\left( t \right),$$

$$s_{i}\left( t \right)=M\left( t \right)V_{i}\left( t \right)-HP_{i}\left( t \right),$$

where the subscript $i$ is the index of the $i$th bank, $S_{i}^{(u)}$ is the Sharpe ratio where a return rate $\bar{r}_{i}^{(u)}(t)$ is calculated every $u$-minute, $N^{(u)}$ is the number of the data points sampled every $u$-minute for a week, $\sigma_{i}^{(u)}$ is the standard deviation of the portfolio value, $s_{i}\left( t \right)$ is the profit value at time $t$, $M\left( t \right)$ is the market mid-price at time $t$, $V_{i}\left( t \right)$ is the trading inventory at time $t$ where buy (sell) volumes are defined to have a plus (minus) sign (i.e., when $i$th bank bought 10 volumes and sell 5 volumes until time $t$, the trading inventory at time $t$ is calculated as 10-5=5 volumes), and $HP_{i}\left( t \right)$ is the cumulative historical prices of inventory until time $t$. We assume the average trading inventory from time $t-u$ to $t$ in yen is calculated using the average of inventories at time $t$ and time $t-u$, which is multiplied by $M\left( t \right)$ for simplicity. Note that, from above calculation, we exclude samples for which the average inventory $\bar{V}_{i}\left( t \right)$ is exactly 0.

In this study, we set $u$ 20, however, this setting does not qualitatively reflect the difference

in the main result as long as $u$ is not too large ($u<30$). For example, the Spearman's rank correlation coefficient between the consumption probabilities and the Sharpe ratios are 0.59 ($u=10$), 0.54 ($u=20$), and 0.42 ($u=30$).
